# Book Reviews

**Published:** 1914-04

**Authors:** 


### BOOK REVIEWS.

---

*Books mentioned may be inspected at and ordered through this office. So far as possible, books received in any month will be reviewed in the issue of the second month following.*

---

Diseases and Deformity of the Foot. John Joseph Nutt, B. L., M. D., Surgeon-in-Chief New York State Hospital for the care of Crippled and Deformed Children; Surgeon Sea Breeze Hospital, etc., etc. $2.75, E. B. Treat & Co.

This book is written, as the author states in his preface, for the use of physicians who have not had the time or the opportunity for a thorough study of the foot. The author has attempted to cover a very large topic in a very limited space, and as is inevitable with a book written in this way, many important subjects are naturally abbreviated, and much that is of value is necessarily omitted.

In the chapter of Anatomy, the supernumerary bones of the foot are but touched upon. The mention and treatment of Rigid Foot, a condition which is so frequently seen following infections, has been entirely overlooked. The chapters on Weak Foot and Flat-foot, however, are, with a few exceptions, of great value as are also the chapters on Infantile Paralysis and Congenital Club-foot. The latter subject, Congenital Club-foot, is treated quite extensively, and shows that the author has had vast experience in this type of deformity.

The book is of value especially because it shows the wide range of conditions affecting the foot, and that the orthopedist, when seeing a foot case, has much more to take into consideration than simply flat-foot in making a proper diagnosis. Some of the more modern procedures are also presented; namely, Heliotherapy, Stoeffel's treatment for spastic paralysis, and many others of equal value.

---

MORTALITY STATISTICS, 1912. Bureau of the Census.

This is the 13th annual report and contains much interesting information. The chart on page 18, showing the curve of death rates from several common diseases, deserves study. Since 1900, tuberculosis has declined, fairly steadily, from a trifle over 2 per 1000 population to a trifle less than 1.5. This represents a close parallelism with the decline of the total mortality and warrants considerable optimism. Typhoid has declined from about 36 to 19 deaths per 100,000 population. The statement so often repeated that pneumonia deaths compensate for any decline in tubercular mortality appears to be unwarranted. On the contrary, there has been a decline from about 18 to 13 deaths per 1000 population. Organic heart disease, nephritis, cancer, apoplexy, all show moderate increase, to be expected as the general death declines and the average longevity increases.

---

DIAGNOSTIC METHODS, Herbert Thomas Brooks, A. B., M. D., Memphis. C. V. Mosby Co., St. Louis. 82 pages; $1.00; 2d edition.

This is a brief compend intended especially for students, but, while it is obviously not an extensive and intensive work, it is well adapted to the use of the general practitioner. It is a good deal better to make a moderately complete routine examination of all cases than to attempt, spasmodically, elaborate methods, or to buy a work beyond one's ability, equipment and time, and to become discouraged with the whole range of modern diagnostics.

Practical Sanitation, Fletcher Gardner, M. D., Health Commissioner of Monroe, Ind., and James Persons Simonds, A. B., M. D., Prof. of Preventive Medicine and Bacteriology, University of Texas. C. V. Mosby Co., St. Louis. 403 pages, illustrated; $4.00.

This work is especially intended for the use of health officers but also for physicians generally. It is to the best of our knowledge, the only book dealing with sanitation from this standpoint. The work is arranged in three parts—epidemiology, general sanitation and laboratory methods. It contains schedules for sanitary service in cities, samples of blanks used by health departments, criticisms of reports of vital statistics, etc. Infectious diseases are classified in groups such as show marked analogies or are likely to demand differential diagnosis (as diphtheria, whooping cough, epidemic parotitis, etc.) Quarantine of different kinds and for different diseases is especially well treated. However, the work is complete and excellent throughout.

---

Stammering and Cognate Defects of Speech, C. S. Bluemel, Boulder Col.

In our review in the January issue, we stated that the second volume dealt with systematic pronunciation exercises. We failed to note that the author expressed a general disapproval of all these systems in the preface. It is so rarely that an author presents in detail a comprehensive review of matter contrary to his own opinion that we trust we shall be pardoned for the oversight. Dr. Bluemel is to be complimented on his broadness of view in this respect, in giving to readers who may dissent from his opinion, the benefit of matter which they would, otherwise, have to obtain elsewhere, while, on the other hand, his criticisms of the methods alluded to, are of value in forming opinion.

---

Gun Shot Injuries. Col. Louis A. Lagarde, M. D., U. S. A. Medical Corps, retired; late Commandant and Prof. of Military Surgery, U. S. A. Medical School. Published under the direction of the Surgeon General and by authority of the Secretary of War, by Wm. Wood & Co., N. Y.; 498 pages, illustrated, $4.

Col. Lagarde's ample experience and opportunities for employing the accumulated experience of the U. S. Army, as well as his training in didactic methods, well qualify him for the treatment of this subject. The technical details of projectiles are thoroughly discussed. It is somewhat surprising to note the variation in the tetanus rate in warfare and that even in military surgery, the blank cartridge is now especially to be feared. X-ray methods are well discussed, especially with regard to portable field apparatus. Many of the illustrations show the ultimate effect of projectiles on bones. Lesions of the soft parts, especially of the viscera, are given due attention.

---

PATHOGENIC MICRO-ORGANISMS. Ward J. MacNeal Ph. D., M. D., Prof. of Pathology and Bacteriology, N. Y. P. G. Medical School and Hospital. (Based upon Williams' Bacteriology). P. Blakiston's Son & Co., Philadelphia; 462 pages, 213 illustrations; $2.25.

This is virtually a sixth edition of the work of Dr. Herbert Upham Williams, Dean of the Medical Department of the University of Buffalo, who wished to be relieved of further responsibility on account of pressure of other duties. The present author has impressed his own personality upon the work, without failing to retain the excellence of the original. It is a thorough treatise, discussing both general bacteriologic technic and the morphology and biology of micro-organisms, including historic notes, references to specificity, diagnostic problems, etc. As Williams' book has been so long used and so favorably received, as noted by the number of editions through which it has passed, further comment is unnecessary.

---

THE INTERVERTEBRAL FORAMEN, An Atlas and Histologic Description of an Intervertebral Foramen and its Adjacent Parts. Harold Swanberg, Chicago. Chicago Scientific Publishing Co.; 101 pages, 16 original full page plates; $3.00.

This is one of the most erudite and interesting monographs on an anatomic subject that has been presented. The author used an eight-months old domestic cat, prepared sections in the most careful manner, and subjected them to histologic examination of the most minute degree.

---

LEADERS IN HOMEOPATHIC THERAPEUTICS. By E. B. Nash, M. D. author of "Leaders in Typhoid," "Regional Leaders," "Leaders in Sulphur," "Leaders in Respiratory Organs," "How to Take the Case," and "Testimony of the Clinic." Fourth edition, 493 pages. Cloth, $2.50 net. Postage, 16c. Philadelphia. Boericke & Tafel, 1913.

This is not a biographic work, as we thought at first glance, but a homeopathic materia medica and therapeutics, the leaders being the important drugs used. It is a genuine, thorough presentation of the subject by a genuine homeopath. We do not feel competent to give a systematic review from this standpoint and certainly are not inclined to subject a carefully written work to criticism from the standpoint of one unfamiliar with the fundamental premises of the author.

---

DIAGNOSIS IN THE OFFICE AND AT THE BEDSIDE. The Use of Symptoms and Physical Signs in the Diagnosis of Diseases. By Hobart Amory Hare· M. D., Professor of Therapeutics, Materia Medica and Diagnosis in the Jefferson Medical College of Philadelphia. New (7th) edition, thoroughly revised and rewritten. Octavo, 547 pages, with 164 engravings and 10 full-page plates. Cloth, $4.00 net. Lea & Febiger, Philadelphia and New York, 1914.

This book is of an eminently practical nature and wisely graded to standards applicable by the clinician. To a considerable degree, illustrative cases are used for didactic instruction, without extending their discussion to the degree appropriate to a clinical lecture. For instance, a cross section (full page plate) shows the actual findings in a case of colloid cancer of the peritoneum and iliac flexure. The sphygmograph and cardiosphygmograph are shown in actual operation. This method, consistently carried out, affords a better grasp and is more impressive than an entirely impersonal description· yet it is employed only to supplement a systematic general discussion of Diagnosis.

---

THE ELEMENTS OF HOMEOPATHIC THEORY, PRACTICE, MATERIA MEDICA, DOSAGE AND PHARMACY. By Drs. F. A. Boricke and E. P. Anshutz. Third Revised Edition. 223 pages. Cloth, $1.00. Postage, 5 cents. Philadelphia. Boericke & Tafel. 1914.

Part I, Generalities, is naturally of most interest to the non-homeopath. A brief biograph of Hahnemann, a list of homeopathic text books, including one on veterinary medicine and a discussion of the general principles of therapeutics, are contained in this section. The symptomatic indication, potency, etc.· of each drug, follows according to an alphabetic arrangement.

---

THE ANATOMIST'S NOTE BOOK. A. Melville Paterson, M. D., F. R. C. S., Liverpool; Oxford University Press, American Branch, 35 W. 32, N. Y,; 350 pages, copiously illustrated. $2.

This gives concise directions for dissection· by regions, each well illustrated by full page cuts and with blank leaves for notes.

Muscles, vessels and nerves, ligaments, etc., are chiefly covered, the book not pretending to enter into the anatomy of the central nervous system or the viscera, except as touched upon in ordinary dissection.

---

THE EARLY DIAGNOSIS OF TUBERCLE. Clive Riviere, M. D., F. R. C. P., London; Oxford University Press, American Branch, 35 W. 32, N. Y.; 260 pages including 31 illustrations; $2.00.

This is divided into parts, for adults and children, the latter including a lucid description of the French conception of "ganglio-pulmonaire." The discussion of temperature, especially with regard to the diagnostic value of rise of temperature on exertion deserves special attention. A negative general and febrile reaction to tuberculin is considered quite reliable, but elevations of temperature occur after its use in a third of normal cases and in half or more of various infections, as well as in tuberculosis. These are random topics, showing the value of the information contained in this book which seems always to be based on facts and on facts not readily available or generally known.

---

INFECTIONS OF THE HAND. A Guide to the Surgical Treatment of Acute and Chronic Suppurative Processes in the Fingers, Hand and Forearm. By Allen B. Kanavel, M. D., Assistant Professor of Surgery, Northwestern University Medical School, Chicago. New (2d) edition thoroughly revised. Octavo, 463 pages, with 147 illustrations. Cloth, $3.75, net. Lea & Febiger, Philadelphia and New York, 1914.

Some time ago we reviewed the first edition of this work. The exhaustion of the first edition has given opportunity for revision and the addition of new matter. The author states in the preface that he has hesitated to enter too much into detail, yet a highly specialized work of this kind requires detail. This book is one of a few which has entered a practically untilled field and has already established itself as the authority in this field.

---

TRANSACTIONS OF THE AMERICAN ASSOCIATION OF G. U. SURGEONS. Vol. 8, covering 27th Annual Meeting at Atlantic City, May 6 and 7, 1913. (Secretary Dr. Richard F. O'Neil, Boston.)

This is a paper-bound work of 313 pages, well illustrated, comprising a number of valuable monographs, lists of officers and members, etc.

Buffalo Historical Society Publications. Vol 17, edited by Frank H. Severance, Secretary; 453 pages.

The permanent value of any similar organization depends mainly on the work of the Secretary. This volume contains reproductions of photographs of the board of managers, including three physicians—G. Hunter Bartlett, A. H. Briggs and Lee H. Smith. Various papers of local interest are contained in this volume, but the most important part of the work from the general historic standpoint is the collected papers of Gen. Sir Roger Hale Sheaffe, who assumed command of the British army on the Niagara Frontier after the death of Gen. Sir Isaac Brock. An interesting medical item is a bill of Dr. Cyrenius Chapin, paid by Erastus Granger for medical attendance on and medicines furnished to Red Jacket—19 calls and various powerful medicines such as several lots of croton oil pills, tartar emetic, Epecac (sic) amounting in all to 1 pound, 17 shillings.

---

Radium Therapeutics. N. S. Finzi, M. B., M. R. C. S., L. R. C. P., L. S. A., London; Oxford University Press, American Branch, 35 W. 32, N. Y.; 112 pages, illustrated; $2.00.

This book includes an excellent systematic treatise on the properties of radium, its disintegration products, emanations, etc., a general description of its action on tissues, micro-organisms, etc., leading up to the detailed consideration of the technic of its application in therapeutics. It is stated that in spite of its limitation to inoperable cases, 10-15 per cent. of cancers disappear and in some cases, no recurrence has occurred for several years. Definite and apparently candid and reliable statements are similarly, made for each special disease treated so that the work is of the utmost value and timely. We doubt, however, whether many of our readers will work with a gram of radium, as the author has done in many instances.

---

We have received a large number of Inaugural Dissertations from the University of Göttingen. These monographs cover a wide range of medical subjects and are at the service of anyone interested.

---

The Clinics of John B. Murphy, M. D., at Mercy Hospital, Chicago. Volume III, Number 1. Octavo of 190 pages, 91 illustrations. Philadelphia and London: W. B. Saunders Company, 1914. Published bi-monthly. Price per year, paper, $8.00; cloth, $12.00.

These Clinics have now entered upon the third annual volume. They continue in the same general style that has been noted previously. There are profuse illustrations, especially of the bone cases. It is stated that the subject of surgical diagnosis will form an important part of subsequent numbers.

---

A STUDY OF NORTH APPALACHIAN INDIAN POTTERY. Christopher Wren, Plymouth, Pa., republished from Vol. 13 of the Proceedings of the Wyoming Historical and Geological Society of Wilkes-Barre by the E. B. Yordy Co., Wilkes-Barre, 1914. 101 pages, illustrated.

This is a valuable study of local archæologic material, containing interesting general observations on pottery and including a description of pipes, steatite vessels and various sites of aboriginal occupancy. Excepting details noticeable only to an expert, there is a marked similarity to the remains found about Buffalo. There is one little point in psychology that may interest the medical reader. Prehistoric Indian pipes, when ornamented at all with anything beyond a conventional device, usually have the ornament—human or animal head, etc.—facing the smoker, whereas, Indian pipes made after the introduction of European ideas, usually followed the latter custom of turning the ornament away from the smoker. This one point has a considerable value in determining the antiquity of a relic. The reason is probably to be found in the solitude of the Indian, who probably gained some sense of companionship from the figure facing him, whereas, the European, less in need of companionship, instinctively seeks to impress the outsider by putting his decorations where they will most readily attract attention.

---

PROGRESSIVE MEDICINE. Vol. 16, No. 1, March 1, 1914. Edited by Drs. Hobart A. Hare and Leighton F. Appleman of Philadelphia, published by Lea & Febiger, Philadelphia; $6.00 per year (quarterly issues.)

The contents of the present volume are: Surgery of the Head and Neck, by Dr. Charles H. Frazier; Surgery of the Thorax, including the Breast, Dr. George P. Müller; Infectious Diseases, including Rheumatism, Pneumonia and Influenza, by Dr. John Ruhräh; Diseases of Children, by Dr. Floyd M. Crandall; Rhinology and Laryngology, by Dr. George B. Wood; Otology, by Dr. Arthur B. Duel. There are 406 pages, well illustrated. It should be remembered that this series is not an index medicus in the ordinary sense, but contains monographs each of which consists of a carefully digested original article in which special attention is given to the progress of medicine in the particular aspect viewed.

Annual Report of the Surgeon General of the Public Health Service, 1913. Government Printing Office, Washington.

This is a book of 318 pages, giving detailed information of the work of the service, even including some of its scientific work.

---

Anatomy and Physiology For Nurses. Amy E. Pope, Instructor in the Presbyterian Hospital School for Nursing, New York; published by G. P. Putnam's Sons, New York and London; 554 pages, 135 illustrations; $1.75.

The plates are clear and many of the illustrations are in color. Diagrammatic representations, as of the Haversian system, have been employed in several instances. While it is a debatable question as to how much technical medical training should be required of the nurse, and while it is obviously impossible in any science to give an absolutely accurate impression without entering into qualifying details in full, it seems to us that the author has made an admirable compromise and has prepared a text book not only adapted to the use of nurses but of high school and college students. We would go further and suggest that a course in which anatomy and physiology are combined, and of about this degree of intensity, might well be employed for medical students as a preliminary to the usual full descriptions. We are inclined to believe that a bird's-eye view of these branches, combined so as to impress on the student the functions as well as the elementary structure and relations of the organs would stimulate his attention to more formal and difficult study.

---

Immunity, Methods of Diagnosis and Therapy and Their Practical Application, by Julius Citron, Berlin, translated by A. L. Garbat of New York; second edition, revised and enlarged; 267 pages, 30 illustrations, 2 colored plates and 8 charts; $3.50. P. Blakiston's Sons & Co., Philadelphia.

The demand for a second edition of this work, after a year, attests the fact that Dr. Citron's work is not botanically related to him. The title clearly indicates the scope of the book, as both a systematic treatise, though not entering into all details of amynologic theory, and a practical laboratory guide.

---

Annual Report of the Directors of the American Telephone and Telegraph Co., New York, 1914, pamphlet, 68 pages.

This report shows the financial strength and usefulness of the Bell System. Beginning with a scarcely noticed exhibit at the

Centennial Exhibition of 1876, this system reached the 100,000 mark in 1884, doubled in 1891 and passed the million mark in 1902. Since the development of competition shortly after this date, and the necessary return to the flat rate in many cities, the increase has been remarkable. At present, over eight million Bell telephones are in use: 1:12 of the population. The prosperity of the company itself, as well as the saving of time and, in many instances of life, to the public, is shown to have been wonderfully increased by the stimulus of competition, while every user can attest the improvement of service.

---

MANUAL OF SURGERY. Alexis Thomson and Alexander Miles of Edinburgh. Published by the Oxford University Press, American Branch, 35 W. 32, New York; Vol. 3, Operative Surgery, 2d edition; 620 pages, 255 illustrations; $3.50.

We have already reviewed other volumes in this series. Each is complete in itself. While the books of this series are small, they cover all of the usual surgical procedures and omit comparatively few rare operations. By employing a brief literary style and avoiding the repetition of details readily understood, they are really of more value to the average surgeon than works which apparently assume that one entirely ignorant is to be guided through the steps of an operation or than those which aim to amass all possible opinions and observations on every operation.

---

DISEASES OF THE HEART. James Mackenzie, M. D., F. R. C. P., LL. D., F. R. C. P. I., London. Oxford Medical Publications, Oxford University Press, American Branch, 35 W. 32, New York. 3d edition; 502 pages, illustrated; $5.50.

This is an unusually large and detailed work for an English author, fulfilling the standards of completeness set by German and American authors. The first edition was published in 1908, the appreciation of its value demanding a second edition in 1910, and the present, rewritten last year. The author calls attention in the preface to three developments which have materially changed the point of view of an author of a book on this subject: 1. The clearer differentiation of signs of disease, especially by the electro-cardiograph. 2. The bearing of heart manifestations on the question of present or remote heart failure. 3. The realization that the physiologic principles of the circulation cannot be based entirely on normal hearts but must be modified according to existing heart lesions. This enunciation of the scope of the work implies a broadness of view and an inclusive significance of heart disease. The appendix reinforces the didactic and systematic treatment of the subject, with clinical illustrations of great value, including 92 case reports, bearing on almost every phase of the general subject.

---

Defective Ocular Movements and Their Diagnosis. E. and M. Landolt, Paris, translated by Alfred Roemmele and Elmore W. Brewerton. Oxford University Press, American Branch, 35 W. 32, New York; 99 pages, 27 illustrations; $2.00.

This monograph begins with a consideration of the anatomy, physiology and optical mathematics of the extrinsic motor apparatus of the eyes. While intended to be thoroughly practical it necessarily enters into many scientific details, including the central lesions concerned in certain forms of paralysis. The tabular review of Diplopia, giving normal appearances for each eye and abnormal pictures obtained from test type and fixing line, is especially good.

---

Lectures on Tuberculosis to Nurses. Olliver (sic) Bruce, M. R. C. S., L. R. C. P., Joint Tuberculosis Officer, County of Essex, England; 134 pages.

Lectures on Medical Electricity to Nurses. J. Delpratt Harris, M. D., M. R. C. S., Royal Devon and Exeter Hospital, England; 88 pages, illustrated.

These two books are published by Paul B. Hoeber, 69 E. 59, New York, at $1.00 each, in a series of monographs for nurses. They give a sufficient understanding of the scientific principles and facts involved and then discuss the respective subjects from the practical standpoint of what is likely to be expected of the nurse in assistance upon actual cases. The authors have avoided the pitfall into which so many have fallen, of entering into too great minutiæ, and this series is destined to be of great value in affording an intelligent comprehension of fundamentals and in impressing the practical duties of the nurse.

---

The Healthy Marriage. G. T. Wrench, M. D., B. S., Past Assistant Master of the Rotunda Hospital, Dublin. Published by Paul B. Hoeber, 69 E. 59, New York; 300 pages; $1.50

This book, dedicated with affection and esteem to the author's mother-in-law, is intended as a "medical and psychologic guide for wives." We like the dedication. Every man who has contracted a eugenic marriage with a woman who is healthy in body and mind ought to recognize a debt to his mother-in-law. In the initial chapter, the writer not only endorses early marriage, but logically recommends parental assistance to that end, when required by the economic difficulties of highly artificial civilization, and draws an analogy with the oriental sense of duty on the part of the parent to provide for the marriage of his sons. In this heresy we cordially agree. We recall, with lasting regret, a brilliant young married woman who died soon after the birth of her second child from exposure and overwork, her relatives in very comfortable circumstances being imbued with the notion that the young people ought to be independent. And we are watching with interest the result of the theory of a prosperous business man that it is cheaper and better to encourage early domestic life in his son than to run the risk of having to pay in something more precious than money for the results of a young bachelor's life.

The author is almost unique among writers who are not professional ethnologists in realizing that savages are almost universally moral and decent. It may interest him, therefore, to know that the reviewer has observed only two Indian relics in western New York that could be considered obscene. One of these is a "maternity" pipe which does not necessarily indicate impurity of thought in its crude designer, and the other was originally an ordinary stone pestle, made obscene by an orthodox Christian white farmer who found and embellished it.

Wrench's book discusses in order the various physician and psychic trials of the married woman and contains much good advice as to the conduct of labor, care of the child, etc.

